# Demographic and Clinical Characteristics of 4556 Type 2 Diabetes Mellitus Patients at a Tertiary Care Hospital in Southern Punjab

**DOI:** 10.7759/cureus.4592

**Published:** 2019-05-03

**Authors:** Ghulam Mohyud Din Chaudhary, Farooq Mohyud Din Chaudhary, Azfar Tanveer, Asim Tameez Ud Din, Sana Mohyud Din Chaudhary, Asma Tameez Ud Din, Aymon Shafi

**Affiliations:** 1 Internal Medicine, Nishtar Medical University & Hospital, Multan, PAK; 2 Gastroenterology, Nishtar Medical University & Hospital, Multan, PAK; 3 Internal Medicine, Rawalpindi Medical University, Rawalpindi, PAK; 4 Internal Medicine, Combined Military Hospital Lahore Medical College and Institute of Dentistry, Lahore, PAK; 5 Dermatology, Nishtar Medical University & Hospital, Multan, PAK

**Keywords:** type 2 diabetes mellitus, hypertension, body mass index, waist circumference, waist to hip ratio, central obesity, socio-demographic and clinical characteristics

## Abstract

Objective

The objective of this study was to analyze the demographic profile of type 2 diabetes mellitus (DM) patients presenting to a tertiary care hospital of Southern Punjab, Pakistan.

Methods

This descriptive study was carried out at the Diabetic Outdoor Nishtar Hospital Multan from 2013 to 2018 after taking approval from the Institutional Ethical Review Committee. All patients were evaluated in detail after obtaining informed consent.

Results

Data of 4,556 patients with type 2 DM were analyzed. There were 2549 (55.9%) female and 2007 (44.1%) male participants in our study. The mean age of our study population was 47.72 years with a standard deviation (SD) of 10.82 years. Seventy-nine percent of the patients belonged to urban areas. Symptoms of polyuria, polydipsia, and polyphagia were found in 72%, 67%, and 59% of patients, respectively. Hypertension was found in 3391 (74%) patients. The mean waist circumference (WC) was 102.85 cm with an SD of 18.14 cm. The mean waist to hip ratio (WHR) was 1.02 with an SD of 0.102. The mean body mass index (BMI) was 26.50 with an SD of 5.57 kg/m^2^. Obesity (BMI >27 kg/m^2^) was found in 1,891 (41.5%) of patients. Central obesity was found in 80.7% and 94.7% of type 2 DM patients according to the WC and WHR cutoff, respectively. Females were more likely to be obese than males in all parameters of obesity. Central obesity was much more common in female diabetics as compared to male diabetics (odds ratio 4 in WHR criteria versus odds ratio 1.8 in BMI criteria for obese).

Conclusion

Diabetes is more prevalent in females than males and especially affects the middle age group. Hypertension and obesity are important comorbid associations of DM. WC and WHR are more reliable indicators of obesity in type 2 DM patients especially in this part of the world. Central obesity was more prevalent in female type 2 DM patients.

## Introduction

Diabetes mellitus (DM) is a heterogeneous group of metabolic disorders, wherein patients have high blood sugar levels. Type 2 DM is characterized by insulin resistance, in which cells do not respond properly to insulin. Diabetes is a global health problem. According to the World Health Organization (WHO), the number of people suffering from DM is more than 400 million. Its prevalence has risen to 8.5% in 2014 in people older than 18 years. Previously, its prevalence was 4.7% in 1980 [1]. Prevalence rates of DM vary considerably amongst different populations and ethnic groups surveyed [2]. Diabetes prevalence was estimated to increase by 67% from 2010 to 2030 [3]. In developing and under-developed countries, approximately five million deaths were attributed to diabetes in 2015 [4].

Pakistan’s majority of people live in rural areas. Rapid urbanization has led to a rise in DM [5]. The second National Diabetes Survey (NDS) Pakistan found diabetes prevalence at 26.3%. The prevalence of type 2 DM in Malaysia, China, and India was noted to be 22.9%, 11.6%, and 7.3% respectively [6-8]. Age greater than 43 years and family history of DM, obesity, hypertension, and dyslipidemia are considered risk factors for diabetes [9].

Nearly 65% of the adult population in the United States is overweight (BMI > 25 kg/m^2^), according to the National Health and Nutrition Examination Survey (NHANES; 1999-2000). Since the previous NHANES III Survey (1988-1994), obesity prevalence has increased from 23% to 31% [10]. Obesity poses a risk for not only diabetes but hypertension as well, which further increases the morbidity of diabetes. Pakistan Health and Research Council 1998, found hypertension prevalence as 33%, but the latest NDS showed hypertension in the community to be 52.6% [9,11]. In short, diabetes is proving to be a considerable challenge to Pakistan. The aim of our study was to evaluate the changing socio-demographic characteristics of patients having DM.

## Materials and methods

This descriptive study was conducted at the Diabetic Outdoor Nishtar Hospital Multan. After taking approval from the local Institutional Ethical Committee of Nishtar Medical College & Hospital Multan, data of patients coming to the Diabetes Clinic was collected from 2013 to 2018. A total of 4556 type 2 DM patients who gave informed consent were included in this study. The study included adults (>18 years) who were either previously diagnosed diabetics or newly diagnosed type 2 DM (FBS >126 mg/dl and RBS >200 mg/dL). Patients less than 18 years of age, or those who had type 1 DM, impaired glucose tolerance, impaired fasting glucose or gestational DM were not included in the study.

Patients were evaluated with detailed history, examination, and laboratory investigations. The demographic and anthropometric characteristics of the patients were recorded through a pre-designed Performa. Body Mass Index (BMI) was calculated by the formula: weight(kg)/height(m^2^). Patients were considered to be hypertensive if the systolic blood pressure (SBP) was >130 mmHg or diastolic blood pressure (DBP) was >80 mmHg [12]. We defined obesity as BMI >27 kg/m^2^ according to BMI cutoff for Asians [13]. Central obesity was defined according to waist circumference (WC; men ≥90 cm and women ≥80 cm) and waist to hip ratio (WHR) cut-offs (men > 0.9, women > 0.8) [14-15]. The gathered data was entered and analyzed in SPSS version 20.

## Results

Our study included a total of 4,556 patients with type 2 DM, of which 56% were females and 44% were males (*p* < 0.0001). The mean age of the study population was 47.72 with standard deviation (SD) of 10.82 years (mean age of females was 47 years, while that of males was 48 years, *p *< 0.05). Table 1 shows the demographic characteristics of type 2 DM patients in our study. The maximum number of patients (40%) belonged to the 41-50 years age group. Most of the patients belonged to the urban residential area (79.3% versus 20.7%, *p *< 0.0001). About three- quarter patients (74.4%) had concomitant hypertension as well. Greater than 60% had classic symptoms of polyuria, polydipsia, and polyphagia (Table 1). The mean body mass index (BMI) was 26.50 kg/m^2^ with an SD of 5.57 kg/m^2^. The mean waist circumference (WC) was 102.85 cm with an SD of 18.14 cm. The mean waist to hip ratio (WHR) was 1.02 with an SD of 0.102.

**Table 1:** Demographic characteristics of type 2 DM patients Total patients *N *= 4556 * BMI, body mass index; ** WC, waist circumference; *** WHR, waist to hip ratio; DM, diabetes mellitus; SBP, systolic blood pressure; DBP, diastolic blood pressure

Characteristics	n (%)
Age (years)	
<40	1322 (29.0)
41-50	1772 (40)
51-60	1078 (23.7)
>60	384 (8.4)
Gender	
Female	2549 (56)
Male	2007 (44)
Residential Area	
Urban	3615 (79.3)
Rural	941 (20.7)
Obesity (BMI^*^ >27 kg/m^2^)	1891 (41.5)
Central Obesity	
WC** Criteria (men > 90cm, women > 80cm)	3677 (80.7)
WHR^***^ Criteria (men > 0.9, women > 0.8)	4313 (94.7)
Hypertension (SBP > 130 or DBP > 80 mmHg)	3391 (74.4)
Symptoms	
Polyuria	3285 (72.1)
Polydipsia	3074 (67.5)
Polyphagia	2704 (59.4)

Obesity (BMI >27 kg/m^2^) was found in more than 40% of participants. Interestingly, central obesity was found in the vast majority of diabetic patients, more than 80% according to the WC criteria and almost 95% according to the WHR criteria (Table 1). This means that central obesity (WC and WHR) is a more reliable indicator of obesity as compared to BMI in type 2 DM patients. Table 2 depicts the association of different parameters of obesity with gender. As can be seen clearly in all cases, the odds of an obese type 2 DM patient belonging to female gender was significantly higher than belonging to male gender (*p *< 0.001). It is also interesting to note that central obesity is much more common in female type 2 DM patients as compared to male patients (odds ratio 4 in WHR criteria versus odds ratio 1.8 in BMI criteria for obese).

**Table 2:** Association of obesity indicators with gender Total patients *N* = 4,556; female = 2,549 and male = 2007; * waist circumference cutoffs (men > 90cm, women > 80cm), ** waist to hip ratio cutoffs (men > 0.9, women > 0.8)

Variables		Gender		Odds Ratio	P value
		Female n (%)	Male n (%)		
Body Mass Index (kg/m^2^)	<18.5	133 (52)	123 (48)	1	<0.001
	18.5-22.9	502 (52.7)	451 (47.3)	1.0	
	23-26.9	738 (50.7)	718 (49.3)	0.95	
	>27	1176 (62.2)	715 (37.8)	1.8	
Waist Circumference^*^ (cm)	Normal	367 (41.8)	512 (58.2)	1	<0.001
	High	2182 (59.3)	1495 (40.7)	2.0	
Waist to Hip Ratio^**^	Normal	62 (25.5)	181 (74.5)	1	<0.001
	High	2487 (57.7)	1826 (42.3)	4.0	

## Discussion

Our study found that DM affects mostly the middle age people and its prevalence is highest in them. In our study, the highest number (40% of the study population) of type 2 DM patients belonged to the 41-50 years age group. This finding was supported by various other studies as well, supporting the fact that DM is a disease of middle-aged adults [16-19]. The female to male ratio of type 2 DM patients in our study was 1.27:1, suggesting that DM is more prevalent in females in this part of the world. This was, however, different from what the WHO Expert Committee on DM found in 1980. They reported a male preponderance in South Asian races. Another study also found male preponderance in DM [20].

Hypertension is very important comorbid of type 2 DM. In our study, 74% of type 2 DM patients had concomitant hypertension. Various studies found the prevalence of hypertension in type 2 DM patients in the range of 33% to 54% [19,21-22]. Our study had a markedly higher prevalence of concomitant hypertension as compared to these studies. Approximately 79% of the patients in our study belonged to urban areas. This was comparable to another study that found 73% of patients belonging to urban areas [19]. The classical symptoms of diabetes such as polyuria, polydipsia, and polyphagia were found in 72%, 67%, and 59%, respectively. These findings were comparable to what another study [19] found.

Obesity is strongly associated with diabetes. Our study population had a very high prevalence of obesity. Approximately 40% of our study population had a high BMI (cut-off BMI >27 kg/m^2^ for obese). A study found elevated BMI in 42% of type 2 DM patients, which was comparable to our study [19]. Another study identified elevated BMI in 21% of type 2 DM patients [22]. A large number of type 2 DM patients in our study had central obesity as well. Approximately 80% had high WC, while almost 95% had high WHR. A study determined central obesity in 58% of type 2 DM patients [19]. However, another study found that 72% of type 2 DM patients had high WHR [22]. Various other studies also found similar results [23-24]. Even though central obesity was quite a frequent finding in different studies around the world, our study had the highest prevalence of central obesity. The odds of a female diabetic patient to be obese were much higher as compared to male diabetics. This observation was much more significant when central obesity was taken into account. This finding was comparable to another study, which found a statistically higher mean BMI in female type 2 DM patients as compared to male [19]. This may be due to the fact that most females are housewives in this part of the world and mostly lead a sedentary lifestyle.

Our study had some limitations. As our study was conducted out at a tertiary care center, it may not be representative of the general population. This was a single center study. Multi-center studies should be undertaken in the future for better comparison among different populations and understanding the changing trends of this disorder in this part of the world. This study was carried out in a government-run hospital, and most of the patients coming to the diabetic clinic belong to low socioeconomic status. Patients from the surrounding private clinics should also be enrolled in future studies.

## Conclusions

DM has become a challenging health issue in this part of the world. Without understanding the clinical and demographic features of the people affected by this condition, we will not be able to control this disorder. Our study showed that DM is more prevalent in females and affects mostly the middle age group. The concomitant illnesses such as hypertension and obesity are very common among the type 2 DM patients of South Punjab. Central obesity as measured by WC and WHR had an alarmingly high prevalence in type 2 DM patients of our study. Female type 2 DM patients were more likely to have central obesity as compared to male type 2 DM patients.
